# Presence and regulation of cannabinoid receptors in human retinal pigment epithelial cells

**Published:** 2009-06-14

**Authors:** Yan Wei, Xu Wang, Ling Wang

**Affiliations:** 1 Department of Ophthalmology, Ruijin Hospital, Shanghai Jiaotong University School of Medicine, Shanghai, People's Republic of China; 2 Drug Discovery and Design Centre, State Key Laboratory of Drug Research, Shanghai Institute of Materia Medica, Chinese Academy of Sciences, Shanghai, People's Republic of China

## Abstract

**Purpose:**

Cannabinoid receptors have been detected in neuron cells and proposed as potential therapeutic agents in neurodegenerative disorders because of their involvement in controlling neural cell survival and death. However, their presence and role in human retinal pigment epithelial (RPE) cells, which play a key role in initiating and developing age related macular degeneration (ARMD), have never been investigated. Here we analyzed the expression of and changes in cannabinoid receptors (CB1 and CB2) and one enzyme responsible for endocannabinoid hydrolysis, fatty acid amide hydrolase (FAAH), in RPE cell oxidative damage process, a cellular model of ARMD.

**Methods:**

Primary human RPE cells and cells from the ARPE-19 cell line were cultured and exposed to H_2_O_2_ for 24 h to induce oxidative damage. Real time RT–PCR, immunofluorescent staining, and western blot methods were performed to study the expression of and changes in CB1 and CB2 receptors, and FAAH. Cell viability and reactive oxygen species (ROS) production were measured by using 3-(4,5-dimethylthiazol-2-yl)-2,5-diphenyl tetrazolium bromide (MTT) and a dichlorofluorescein (DCF) assay, respectively. PI3K/Akt and ERK1/2 protein expression and activation of signaling molecules were assessed by western blot analysis.

**Results:**

By using real time RT–PCR, immunofluorescent staining and western blot methods, we showed that human RPE cells express *CB1*, *CB2*, and *FAAH*. Meanwhile, oxidative stress can upregulate CB1 and CB2 receptor expression, and downregulate FAAH expression. The CB1/CB2 receptor agonist, CP55,940, and the CB2 receptor agonist, JWH015 significantly protected RPE cells from oxidative damage. In addition, CP55,940 significantly reduced the levels of intracellular ROS, strengthened oxidative stress-induced activation of PI3K/Akt and reduced activation of the ERK1/2 signal pathway.

**Conclusions:**

The results demonstrate the expression and regulation of CB1 and CB2 receptors and FAAH in human RPE cells. The modulation of cannabinoid receptor tone warrants consideration for future therapeutic strategies of ARMD.

## Introduction

The endocannabinoid system (ECS), a new intercellular communication network, has been shown to play a crucial role in neurodegenerative disorders' pathogenesis [1,2]. The ECS consists of the following: the cannabinoid receptors, which include Cannabinoid receptor 1 (CB1)/CB2 receptors and non-CB1/CB2 receptors; the endogenous ligands, primarily anandamide (AEA) and 2-arachidonylglycerol (2-AG); the enzymes responsible for the endogenous ligands' degradation, notably fatty acid amide hydrolase (FAAH), and biosynthesis; and the specific uptake mechanisms [3].

Both cannabinoid receptors subtypes, CB1 and CB2, are abundantly expressed in neurons and can protect neurons from oxidative damage [4]. Cannabinoid receptor induction of neuroprotection has been observed in animal models of some neurodegenerative disorders, including Alzheimer disease [5], Huntington’s disease [6], multiple sclerosis [7], and Parkinson disease [8]. However, the presence and regulation of cannabinoid receptors in retinal neurodegenerative disorders have been studied far less than those in brain and other organ systems.

Age-related macular degeneration (ARMD) is a late-onset neurodegenerative retinal disease, which predominates in the elderly as a cause of irreversible and profound vision loss [9]. ARMD shares many common clinical and pathogenetic properties with other neurodegenerative disorders. Currently, there is no ideal therapy to prevent or cure ARMD. Intake of antioxidants is the only established treatment for those with the atrophic or “dry” form of ARMD [10]. Although treatment options for those with the neovascular or “wet” form have increased recently, even these treatments are largely ineffective for reversing existing visual impairment [11]. Thus, the development of strategies for preventing ARMD assumes great importance; and new treatment approaches should focus on the initial insults that lead to the disease's progression. ARMD initial pathogenesis includes degeneration, dysfunction, or loss of RPE cells caused by oxidative injury [12].

Previous studies have demonstrated the presence of endocannabinoids (AEA and 2-AG) in retinas by gas chromatography and mass spectrometry [13-15]. CB1 receptor, a cannabinoid receptor enriched in neuronal tissue, was localized in the inner and outer plexiform layers of a human retina [16]. Unlike CB1 receptor, CB2 receptor and FAAH were undetectable in the human retina, but were found in rat retina, mostly in the ganglion cells and in the soma of dopamine amacrine cells and large cells [17,18]. The function of ECS in the eye, in addition to regulating photoreception and neurotransmission in the retina, and the effects on intraocular pressure and ocular blood vessels [19-24], also encompasses neuroprotective effects against retinal neurotoxicity [25,26]. This background suggests a functional ECS in the retina. Eyes of patients with ARMD have been show increase levels of endocannabinoids (AEA) in their retina [27]. Therefore, we hypothesized that ECS, acting as a novel signaling system in the retina, may play a substantive role in the ARMD pathophysiological process. Because most effects of endocannabinoids are mediated by binding to cannabinoid receptors and terminated by degradation enzyme FAAH [28]. Thus, the present study aims to examine the expression of CB1 and CB2 receptors, and FAAH in human RPE cells, and their changes in oxidative stress conditions, using a cellular model of ARMD [29]. Our findings may set the basis for the potential pharmacological modulation of cannabinoid receptor tone as a novel therapeutic target in ARMD.

## Methods

### Primary human RPE cells and ARPE-19 cell line culture

Human RPE cells were obtained from eye bank donor eyes, which were cut across the posterior pole, and the vitreous and neural retina were removed. Eyes were obtained from three male human donors between 30 and 50 years of age. None of the donors had a known history of eye disease. The eyes at time of receipt were all integral. RPE cells were isolated within 4 to 16 h after death.The remaining eyecup was washed with phosphate buffered saline (PBS, 8.00 g NaCl, 0.20 g KCl, 0.24 g KH_2_PO_4_ and 1.44 g Na_2_HPO_4_ in 1 l distilled water, pH 7.4) and incubated with 0.025% trypsin-ethylene diamine tetraacetic acid (EDTA; Invitrogen-Gibco, Carlsbad, CA) in a humidified chamber at 37 °C. The cells then were scraped gently and seeded in Dulbecco’s modified Eagle’s medium (DMEM; Invitrogen-Gibco) containing 15% fetal bovine serum (FBS; Invitrogen-Gibco).

A human retinal pigment epithelial cell line (ARPE-19) was purchased from American Type Culture Collection (ATCC; Manassas, VA). The cells were maintained in DMEM/F12 medium (Invitrogen-Gibco) supplemented with 10% FBS (Invitrogen-Gibco) and 1% penicillin and streptomycin, and were cultured at 37 °C in a humidified atmosphere of 95% air and 5% CO_2_. The medium was changed every two days. The ARPE-19 cells were used within 10 generations. Since cells of lower generations were quite sensitive to oxidative stress.

### Cell treatment

The ARPE-19 cells were seeded in flat-bottomed microculture 96 well plate (15,000 cells/well) and allowed to adhere for 24 h. Dose–response assays were performed on the ARPE-19 cells to determine the 50% inhibiting concentration (IC_50_) of hydrogen peroxide (H_2_O_2_). The cells were treated with 0 to 500 μM H_2_O_2_ in serum-free and phenol-free DMEM-F12 medium for 24 h. The 30% H_2_O_2_ stock solution was used within its three-month expiration limit. Working solutions of H_2_O_2_ were made fresh and added to the medium.

CP55,940 (Sigma, St. Louis, MO) is a nonselective CB1 and CB2 cannabinoid receptor agonist. ACEA (Tocris Bioscience, Ellisville, MO) is a selective CB1 agonist. JWH015 (Cayman, Ann Arbor, MI) is a selective CB2 receptor agonist. ARPE-19 cells were preincubated with various concentrations of CP55,940, ACEA, and JWH015 for 15 min before exposure to 200 μM H_2_O_2_ for 24 h in serum-free, phenol-red-free DMEM and F12 media at 37 °C.

For each concentration of H_2_O_2_ and the compounds, five wells were analyzed. Each experiment was performed at least three times.

### RNA extraction

Total RNA was isolated from primary human RPE cells, ARPE-19 cells and, H_2_O_2_ (200 μM)-treated ARPE-19 cells using the RNeasy Total RNA System (RNeasy Mini Kit, Qiagen, Valencia, CA) following the manufacturer's recommendation, and then treated with RNase-free DNase I to remove any contaminating genomic DNA. The isolated RNA had optic density (OD) 260/280 ratios greater than or equal to 2.0 [30]. To synthesize a cDNA template for PCR, we reverse transcribed 1 μg of total RNA with oligo-(dT) primer and reverse transcriptase (ReverTra Ace, Toyobo Co., Ltd., Osaka, Japan). The quality of first-strand cDNA was confirmed by PCR with *β-actin* primers.

### Real-time reverse transcription-polymerase chain reaction (Real-time RT–PCR)

Real-time RT–PCR was performed for quantitative analysis according to the standard protocol using the SYBR Green PCR Master Mix (Toyobo Co., Ltd., Osaka, Japan). PCR conditions for CB1 and CB2 were as follows: after initial denaturation at 95 °C for 5 min, 40 cycles were performed at 94 °C for 30 s, 58 °C for 30 s, and 72 °C for 1 min, followed by a 10 min extension at 72 °C. For FAAH, the conditions were as follows: after initial denaturation at 95 °C for 5 min, 40 cycles were performed at 94 °C for 30 s, 62 °C for 30 s, and 72 °C for 1 min, followed by a 10 min extension at 72 °C.

Primers used were shown in Table 1. Quantification analysis of *CB1*, *CB2*, and *FAAH* mRNA was normalized with *β-actin* as reference. The specificity of PCR amplification products was checked by performing a dissociation melting curve analysis. Relative multiples of changes in mRNA expression were determined with the relative comparative threshold (CT) method.

**Table 1:** Primer sequences used for real time RT–PCR.

**Gene**	**Primer (5′-3′)**
*CB1*	F: TTCCCTCTTGTGAAGGCACTG
	R: TCTTGACCGTGCTCTTGATGC
*CB2*	F: TTTGCTTTCTGCTCCATGCTG
	R: TTCTTTTGCCTCTGACCCAAG
*FAAH*	F: GCCTGGGAAGTGAACAAAGGGACC
	R: CCACTACGCTGTCGCACTCCGCCG
*β-actin*	F: GATGAGATTGGCATGGCTTT
	R: GAGAAGTGGGGTGGCTT

### Immunofluorescent staining

CB1 and CB2 expression in the ARPE-19 cells was determined by immunofluorescent staining. In brief, ARPE-19 cells were grown to confluence in chamber slides (Nalgene-Nunc, Lab-Tek, Naperville, IL). The growth medium was aspirated and the cells were washed three times with PBS, and then fixed with 4.0% paraformaldehye for 20 min at 4 °C. After the cells had been washed with PBS, they were permeabilized with 0.2% Triton X-100 in PBS for 15 min at room temperature. Subsequently, CB1 and CB2 expression in the ARPE-19 cells was determined by immunofluorescent staining using a 1:100 dilution of anti-CB1 (rabbit polyclonal, Abcam, Cambridge, UK) or anti-CB2 (rabbit polyclonal, Abcam), respectively, for 6 h at 4 °C. After the cells had been rinsed with PBS, they were probed with 1:250 goat anti-rabbit FITC (Pierce, Rockford, IL) for 1 h at room temperature. Their nucleus was counterstained with 4’,6-diamidino-2-phenylindole (Molecular Probes, Invitrogen-Gibco, Carlsbad, CA). Images were obtained with a fluorescent microscope (Olympus IX 81) at 20X objective with 1.5X optical zoom (Olympus IX 81, Olympus Optical, Tokyo, Japan).

### MTT assay for cell viability

An 3-[4,5-dimethylthiazol-2-yl]-2,5-diphenyl tetrazolium bromide (MTT) assay is a qualitative index of cell viability. Mitochondrial and cytosolic dehydrogenases of living cells reduce the yellow tetrazolium salt (MTT) to produce a purple formazan dye that can be detected spectrophotometrically [31]. After ARPE-19 cells were preincubated with various concentrations of CP55,940, ACEA, and JWH015 for 15 min followed by exposure to 200 μM H_2_O_2_ for 24 h. Then, MTT (Sigma, St. Louis, MO) was added to a final concentration of 0.5 mg/ml and incubated for 4 h at 37 °C. The culture medium was then removed and the remaining blue precipitate was solubilized in dimethyl sulfoxide, followed by reading absorbance at 570 nm in a plate reader using 630 nm as a reference (Spectra Max 340; Molecular Devices, Sunnyvale, CA). This reading was divided by the adjusted absorbance reading of untreated cells in control wells to obtain the percentage of cellular survival.

### Reactive oxygen species determination

The intracellular reactive oxygen species (ROS) level is an important biomarker for oxidative stress. An increased ROS level generally indicates increased oxidative stress. Relative ROS production was determined by the formation of a fluorescent Dichlorofluorescein (DCF) compound on oxidation of the nonfluorescent, reduced DCF-DA [32]. Cells were incubated with 10 μM DCF-DA at 37 °C for 30 min, and then washed twice with PBS. Relative fluorescence was measured using a fluorescence plate reader at 485 nm excitation and 535 nm emission wavelength (Wallace, Perkin-Elmer, Warrington, UK).

### Western blot analysis

We plated the ARPE-19 cells into six-well plates (150,000 cells/ml). To evaluate the expression of CB1 and CB2 receptors and FAAH, we treated the cells with 200 μM H_2_O_2_ in serum-free and phenol-free DMEM and F12 medium for 24 h. As for the expression of PI3K/Akt and ERK1/2 protein, the cells were pretreated with or without 1 μM CP55,940 for 15 min and then exposed to 200 μM H_2_O_2_ for 24 h. After the treatment, the cells were rinsed twice with ice-cold PBS, then scraped into cell lysis buffer and centrifuged at 13400x g for 10 min at 4 °C. Protein levels were determined using the bicinchoninic acid (BCA) protein assay (Pierce). Next, 15 μg of total protein were solubilized in 2% sodium dodecyl sulfate (SDS) sample buffer, separated on 10% SDS-polyacrylamide gel by electrophoresis (SDS–PAGE) and transferred to nitrocellulose membranes by electroblotting. The blots were washed in Tris-buffered saline containing 0.1% Tween-20 and 5% nonfat dairy milk, and incubated in antibodies to 1:1000 dilution of rabbit polyclonal CB1 and CB2 receptors antibodies (Abcam), 1:1,000 dilution of mouse polyclonal FAAH antibody (Abcam), 1:3,000 dilution of rabbit polyclonal PI3K/Akt and ERK1/2 antibodies (Cell Signaling Technology, Beverly, MA), and 1:10,000 dilution of mouse monoclonal GAPDH antibody (Cell Signaling Technology) at 4 °C overnight. Blots were washed three times, incubated with 1:3,000 horseradish peroxidase (HRP)-conjugated goat anti-rabbit IgG (Pierce) or 1:20,000 HRP-conjugated goat anti-mouse IgG (Pierce) and developed using chemiluminescence (SuperSignal West Pico Luminescent; Pierce) according to the manufacturer’s instructions.

### Statistical analysis

Data were presented as the mean±standard error of the mean (SEM) of the results of two or three separate experiments, as specified in the figure legends. Data were analyzed using ANOVA or a Student’s *t*-test with the SPSS software. A p-value <0.05 was considered significant.

## Results

### Effect of H_2_O_2_ on ARPE-19 cell viability

**Figure 1:**
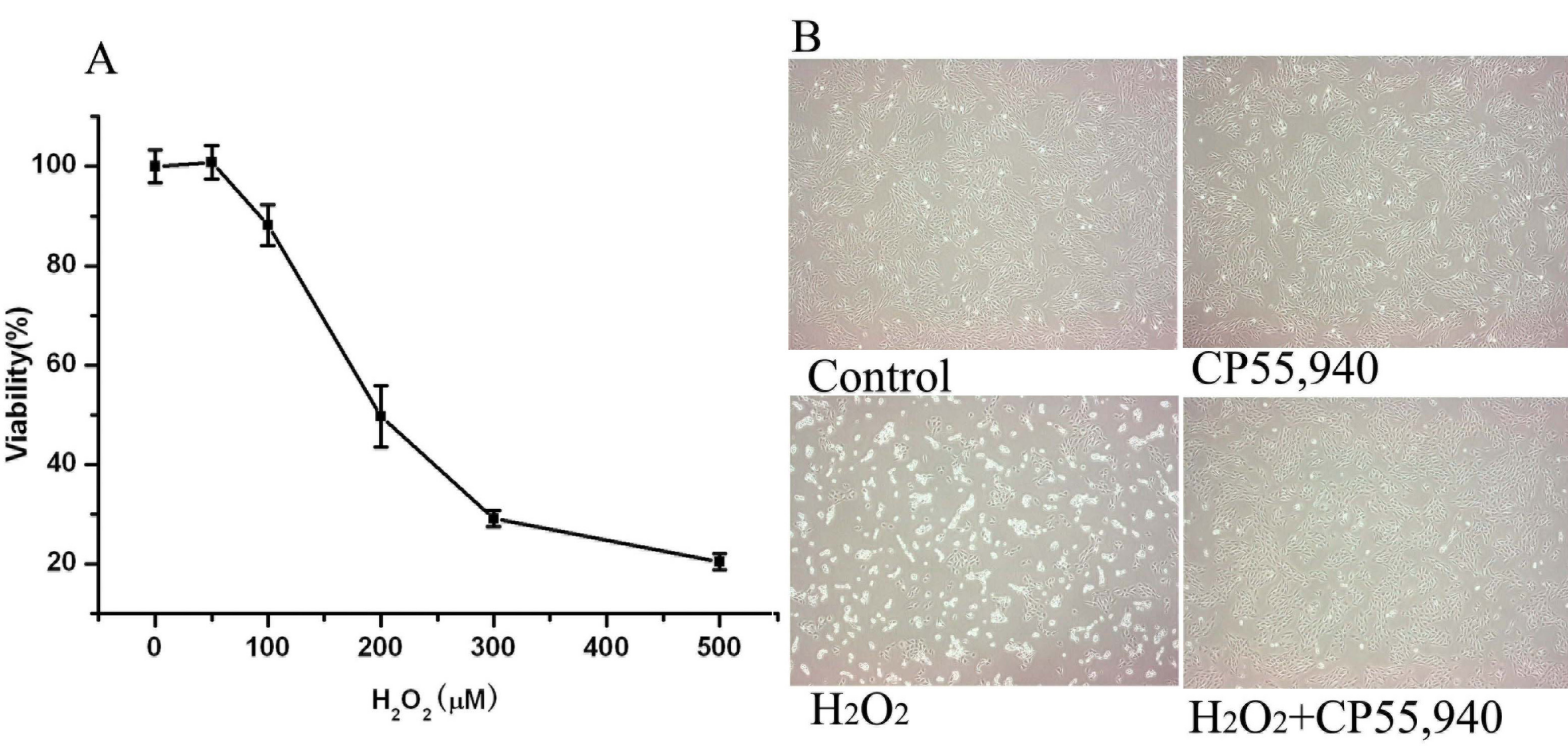
Effect of H_2_O_2_ on cell viability and morphological alterations. **A:** The ARPE-19 cells were incubated with concentrations of 0–500 μM H_2_O_2_ for 24 h. Cell viability was determined by an MTT assay. **B:** Phase contrast photomicrographs of ARPE-19 cells. ARPE-19 cells were treated with 0, 200 μM H_2_O_2_, 1 μM CP55,940, 1 μM CP55,940 plus 200 μM H_2_O_2_ for 24 h (Magnification: 100X).

The MTT assay for cell viability was used to quantify the ARPE-19 cell cytotoxic response to H_2_O_2_. H_2_O_2_ produced a progressive, cytotoxic effect in the ARPE-19 cells, beginning at a dose of 100 μM, with 5.7% cytotoxicity and reaching a maximum toxicity at 77.5% damaged cells measured at 500 μM after 24 h of incubation (p<0.05; Figure 1). The results indicate that treating ARPE-19 cells with H_2_O_2_ for 24 h caused a dose-dependent decrease in their viability with the half maximal inhibitory concentration (IC_50_) of 200 μM.

### Expression of and changes in cannabinoid receptors and FAAH in RPE cells

**Figure 2:**
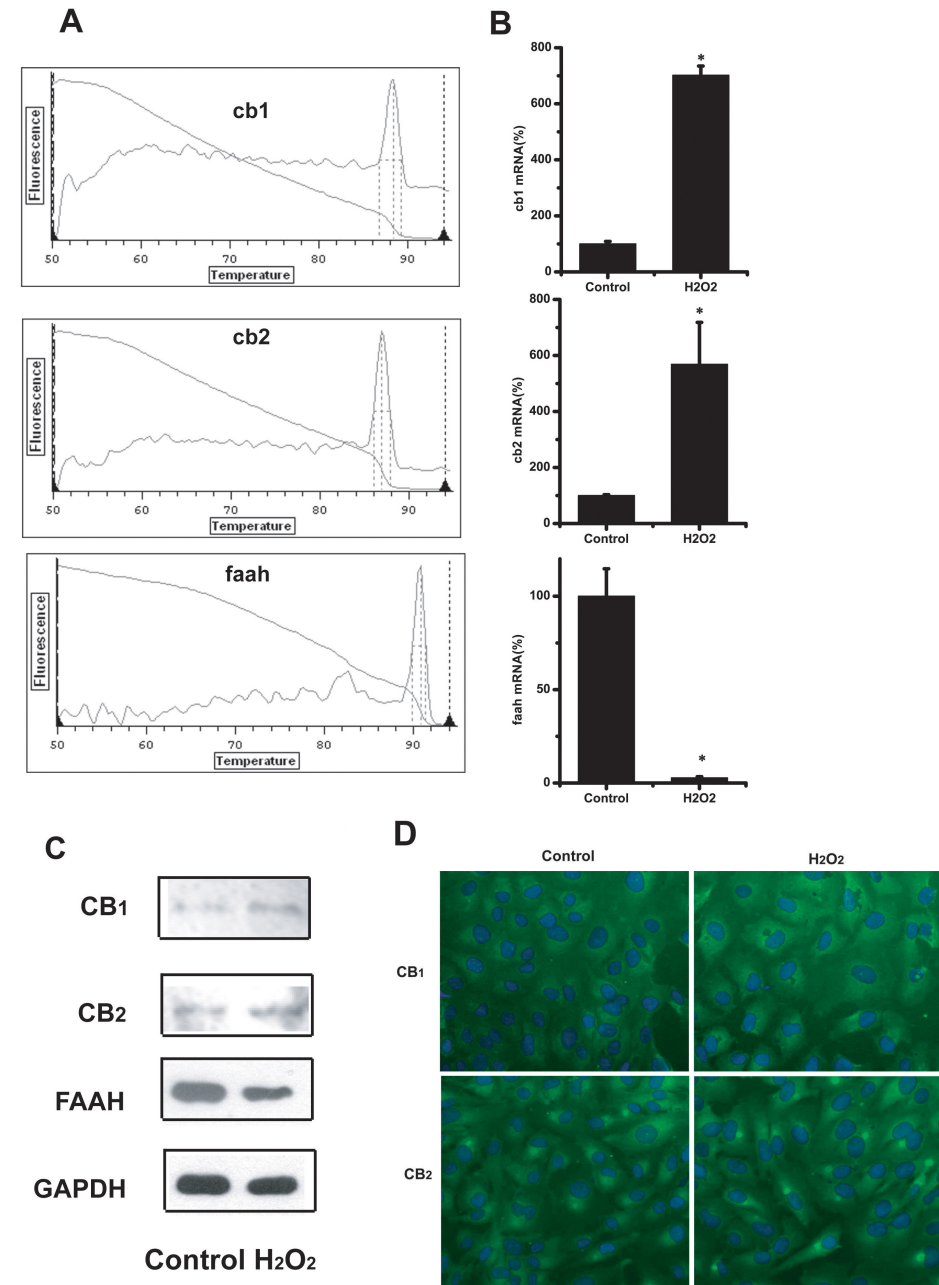
Expression of *CB1, CB2*, and *FAAH* mRNA in human primary RPE cells and changes of *CB1, CB2, FAAH* mRNA and protein expression in 200 μM H_2_O_2_-treated ARPE-19 cells compared to untreated ones. **A:** Expression of *CB1, CB2,* and *FAAH* mRNA expression in human primary RPE cells assayed by real time RT–PCR method. **B:** Changes of *CB1, CB2,* and *FAAH* mRNA expression in ARPE-19 cells assayed by real time RT–PCR method. Asterisk (*) represents the correlation significant at the p<0.05 level and suggest a significant increase or decrease in mRNA expression as compared to control group. **C:** Changes of *CB1, CB2*, and *FAAH* protein expression in ARPE-19 cells assayed by western blot method. **D:** Changes of *CB1, CB2* protein expression in ARPE-19 cells demonstrated by immunofluorescent staining.

As Figure 2A shows, real time RT–PCR revealed primary human RPE cells expressed *CB1*, *CB2*, and *FAAH* mRNA. ARPE-19 cells were treated with 200 μM H_2_O_2_ for 24 h, and the changes in *CB1*, *CB2*, and *FAAH* mRNA expression were determined by quantitative RT–PCR. The results showed that H_2_O_2_-treated ARPE-19 cells had a 7.02 fold increased *CB1* mRNA expression, a 5.68 fold increased *CB2* mRNA expression, and a 35.7 fold decreased *FAAH* mRNA expression, compared to untreated cells (Figure 2B). Consistent with the results of RT–PCR, CB1 and CB2 protein expression increased and FAAH protein expression decreased in 200 μM H_2_O_2_ -treated ARPE-19 cells (Figure 2C). Similar results were obtained with immunofluorescence assays (Figure 2D).

### CP55,940 attenuates H_2_O_2_-induced cytotoxicity and ROS generation in ARPE-19 cells

**Figure 3:**
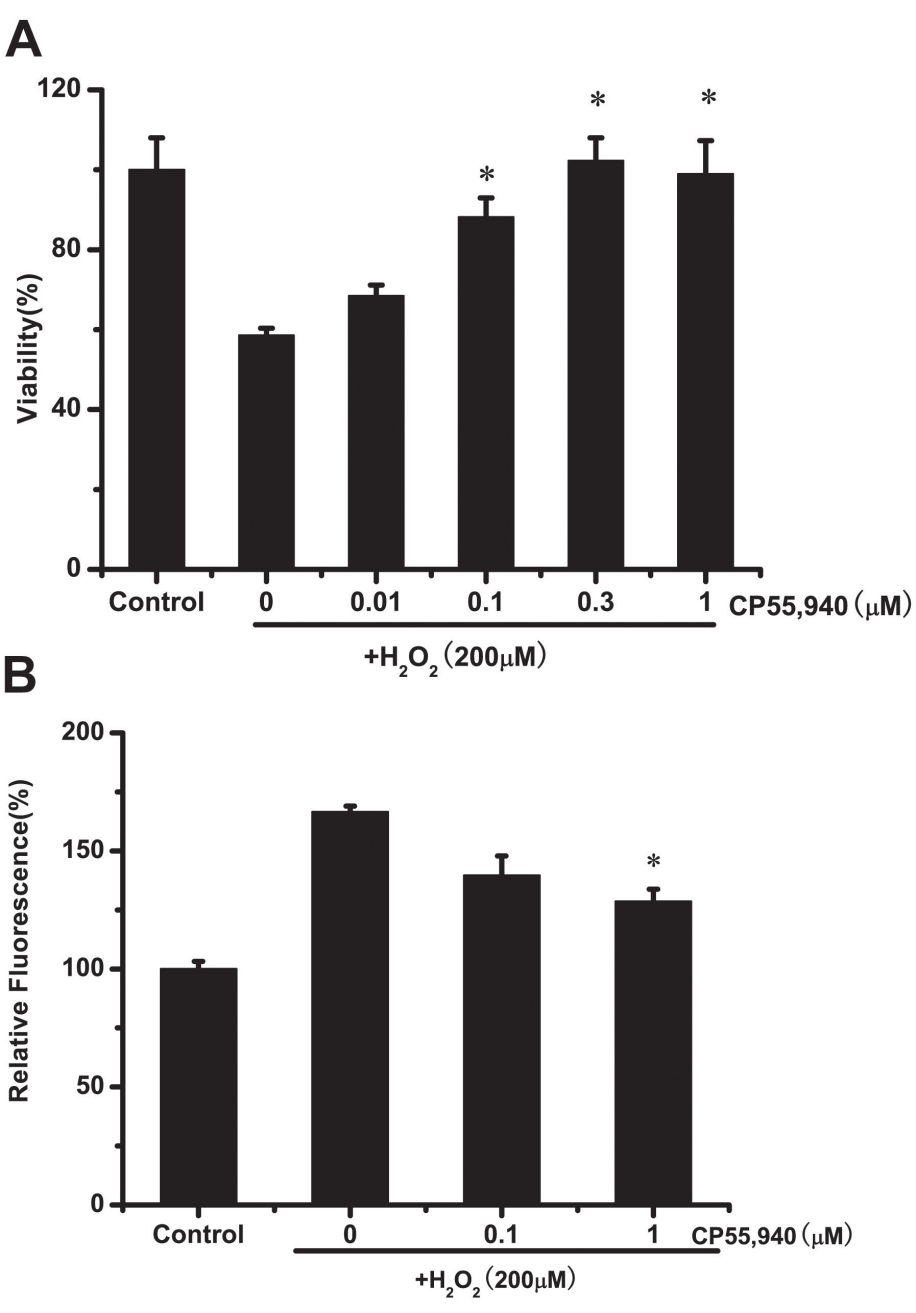
CP55,940 attenuates H_2_O_2_-induced cytotoxicity and ROS generation. **A:** Protective effects of CP55,940 on H_2_O_2_-induced decrease in cell viability measured by the MTT assay. Cells were pretreated with 0–1 μM CP55,940 for 15 min before exposure to 200 μM H_2_O_2_ for 24 h, and cell viability was measured by MTT assay. Asterisk (*) the correlation significant at the p<0.05 level and suggest a significant increase in cell viability as compared to H_2_O_2_-treated group. **B:** Protective effects of CP55,940 on H_2_O_2_-induced increase in intracellular ROS in ARPE-19 cells. Cells were pretreated with 0–1 μM CP55,940 for 15 min before exposure to 200 μM H_2_O_2_ for 24 h, and intracellular ROS was measured by the DCF-DA assay. Aterisk (*) represents the correlation significant at the p<0.05 level and suggest a significant decrease in intracellular ROS generation as compared to H_2_O_2_-treated group.

As Figure 3A shows, 200 μM H_2_O_2_ (24 h) induced a significant (42%) decrease in ARPE-19 cell viability. Pretreating ARPE-19 cells with CP55,940 for 15 min significantly protected them against H_2_O_2_-induced toxicity at concentrations of 0.1, 0.3, and 1 μM, with, respectively, 88%, 98%, and almost 100% of the control. Treatment with H_2_O_2_ at 200 μM for 24 h induced a significant increase in intracellular ROS, approximately 1.7 times compared to the control. Pretreatment with 1 μM CP55,940 for 15 min significantly inhibited ROS generation (23% reduction; Figure 3B). ARPE-19 cells treated with 0, 0.01, 0.1, and 1 μM CP55,940 alone showed no significant effect on viability compared to the untreated control cells.

### CP55,940 enhanced H_2_O_2_-induced activation of PI3K/Akt and reduced activation of ERK1/2

**Figure 4:**
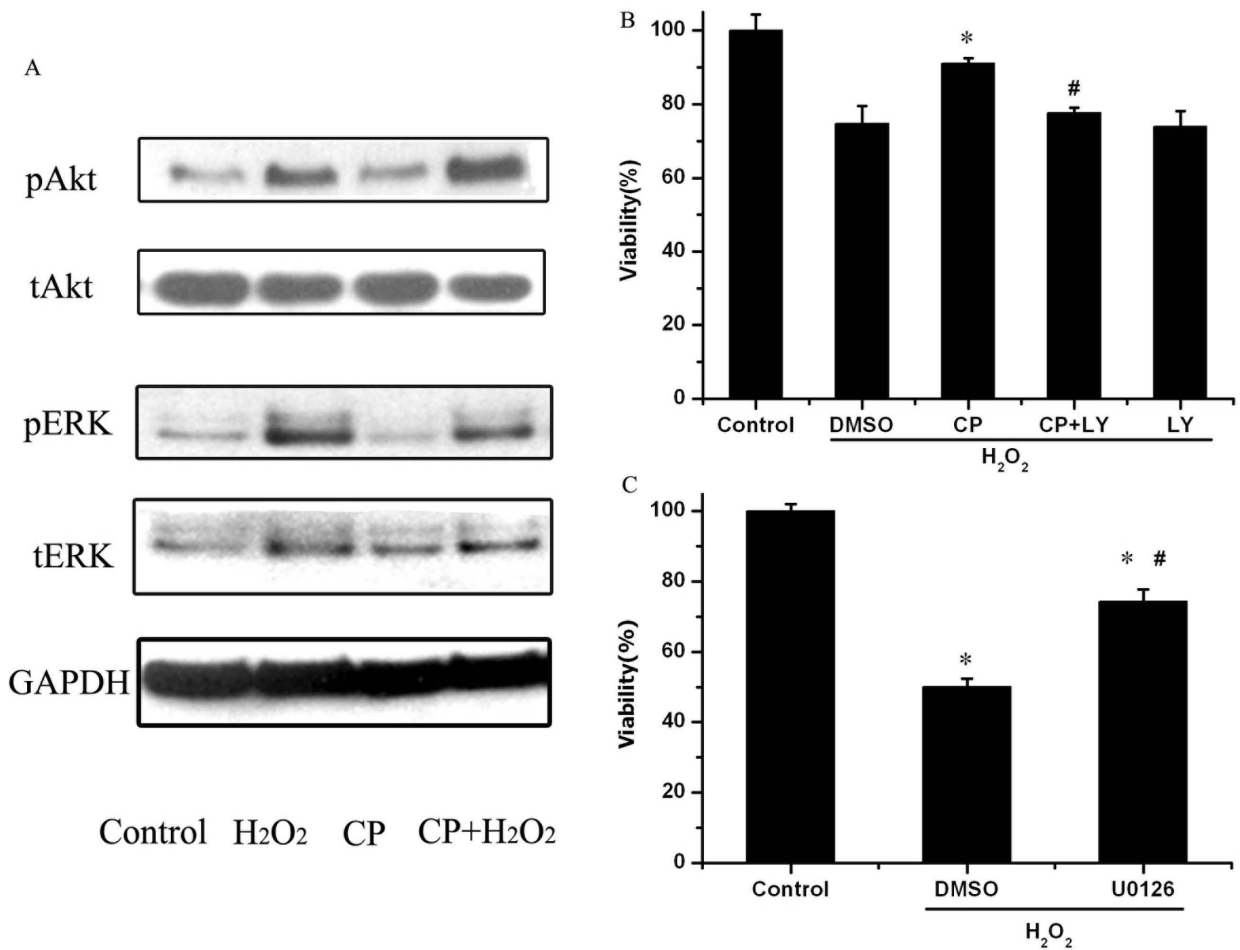
CP55,940 modulates phosphorylation of PI3K/Akt and ERK1/2. **A:** Representative western blot analysis shows that 1 μM CP55,940 enhanced H_2_O_2_-induced activation of p-PI3K/Akt and reduced activation of p-ERK1/2 in ARPE-19 cells. **B:** Protective effects of PI3K/Akt inhibitor (LY294002) on H_2_O_2_-induced decrease in cell viability measured by the MTT assay. ARPE-19 cells were pretreated with or without 20 μM LY294002 for 15 min in the presence or absence of 1 μM CP55,940 before exposure to 200 μM H_2_O_2_ for 24 h. Asterisk (*) represents the correlation significant at the p<0.05 level and suggest a significant increase in cell viability as compared to H_2_O_2_-treated group. Hash mark (#) represents the correlation significant at the p<0.05 level and suggest a significant decrease in cell viability as compared to CP55,940+H_2_O_2_-treated group. **C:** Protective effects of selective ERK1/2 inhibitor (U0126) on H_2_O_2_-induced decrease in cell viability measured by the MTT assay. ARPE-19 cells were pretreated with 20 μM U0126 for 15 min before exposure to 200 μM H_2_O_2_ for 24 h Asterisk (*) represents the correlation significant at the p<0.05 level and suggest a significant decrease in cell viability as compared to control group. Asteriks and hash mark (#) represents the correlation significant at the p<0.05 level and suggest a significant increase in cell viability as compared to H_2_O_2_-treated group. Data are expressed as mean±SEM of results in three separate experiments, each experiment performed in duplicate.

To address the potential role of PI3K/Akt in mediating CP55,940 protection of ARPE-19 cells from oxidative injury, we assessed phosphorylation of PI3K/Akt by western blot analysis. The results showed that PI3K/Akt is activated by H_2_O_2_. Pretreating ARPE-19 cells with 1 μM CP55,940 followed by 200 μM H_2_O_2_ enhanced PI3K/Akt activity compared to cells treated with 200 μM H_2_O_2_ alone (Figure 4A). LY 294002, a specific inhibitor of PI3K/Akt, was used to block PI3K/Akt activation. ARPE-19 cells were pretreated with 20 μM LY294002 for 15 min in the presence or absence of CP55,940, followed by an H_2_O_2_ challenge for 24 h. As Figure 4B shows, inhibition of PI3K/Akt abrogated CP55,940 protection of ARPE-19 cells from oxidative injury.

U0126, a specific inhibitor for the ERK1/2 pathway, was used to evaluate whether ERK1/2 is involved in H_2_O_2_-induced ARPE-19 cell oxidative damage. ARPE-19 cells were pretreated with the selective inhibitor for ERK1/2 before exposure to H_2_O_2_, and cell viability levels were determined. Viability levels increased significantly in the presence of 20 μM U0126 (Figure 4C). Because of that, their phosphorylated ERK1/2 (p-ERK1/2) level was examined by western blot analysis, which showed that ERK1/2 was activated by H_2_O_2_; pretreating ARPE-19 cells with CP55,940 reduced p-ERK1/2 activity, compared to cells treated with H_2_O_2_ alone (Figure 4A).

### CB2 receptor agonist attenuates H_2_O_2_-induced cytotoxicity in ARPE-19 cells

**Figure 5:**
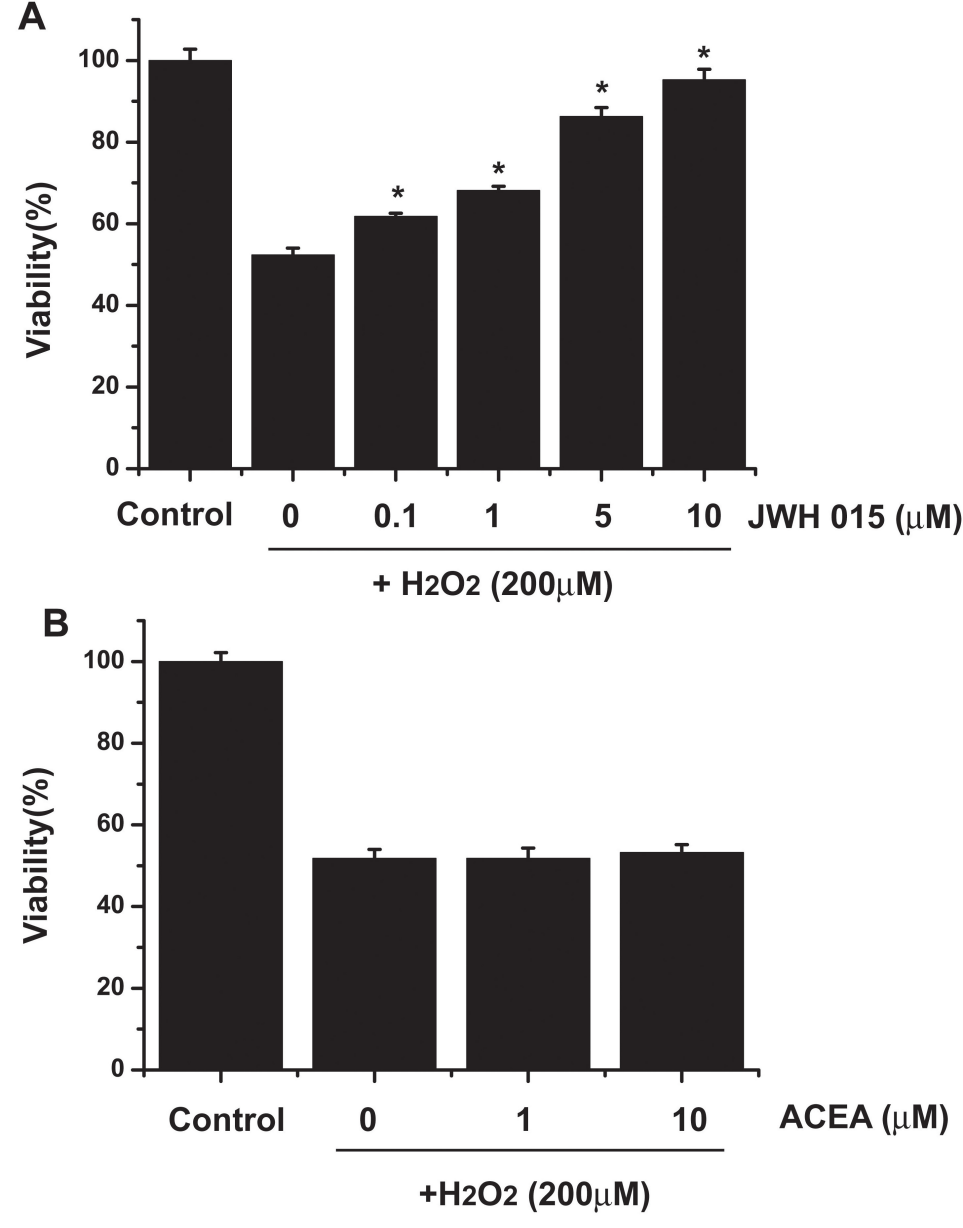
JWH015, but not ACEA, attenuates H_2_O_2_-induced cytotoxicity. **A:** Inhibition of H_2_O_2_-induced decrease in ARPE-19 cell viability by JWH015. ARPE-19 cells were pretreated with 0–10 μM JWH015 for 15 min before exposure to 200 μM H_2_O_2_ for 24 h, and their viability was measured by the MTT assay. Asterisk (*) represents the correlation significant at the p<0.05 level and suggest a significant increase in cell viability as compared to H_2_O_2_-treated group. **B:** ACEA showed no significant effect on H_2_O_2_-induced decrease in cell viability. ARPE-19 cells were pretreated with 0–10 μM ACEA for 15 min before exposure to 200 μM H_2_O_2_ for 24 h, and their viability was measured by MTT assay.

As Figure 5 shows, pretreating ARPE-19 cells with JWH015, a CB2 receptor agonist, for 15 min resulted in a significant protection against H_2_O_2_-induced toxicity at concentrations of 0.1, 1, 5, and 10 μM, with, respectively, 62%, 68%, 86%, and 95% of the control. Pretreatment with CB1 receptor agonist (AECA) for 15 min showed no cytoprotective effect (Figure 5).

## Discussion

To the best of our knowledge, this is the first demonstration of the presence of CB1, CB2, and FAAH in human RPE cells, which play a key role in ARMD initiation and development. Plenty of evidence in the previous literature suggests that oxidative stress is a contributing factor to RPE dysfunction in ARMD [33]. The results led us to further analyze the cannabinoid receptors and FAAH expression changes in RPE cells induced by oxidative stress. Most studies have used human RPE cells as the in vitro study model of ARMD, either primary or cell line. One of the drawbacks of using primary RPE cells is that it provides a limited number of cells and may lack a consistent cellular background. The ARPE-19 cell line overcomes this problem while maintaining the morphological and functional characteristics of primary cells. In addition, ARPE-19 cells respond to oxidative stress (H_2_O_2_, media starvation, etc.) in a fashion similar to that of primary cultured human RPE cells. Therefore, we have used the ARPE-19 cell line for most of our studies. H_2_O_2_ added to the culture medium was used as a chemical oxidant. This agent is convenient and biologically relevant, especially for the RPE cells. H_2_O_2_ has been found in ocular tissues in vivo [34] and can be produced by the RPE cells as a reactive oxygen intermediate during photoreceptor outer segment phagocytosis [35]. For these reasons, we selected H_2_O_2_ for our studies and performed a series of dose response assays to determine the working concentration that led to a consistent and reliable cytotoxicity.

By using real-time RT–PCR and the western blot method, we showed that oxidative stress can upregulate *CB1* and *CB2* receptor expression and downregulate *FAAH* expression in a cell model of ARMD. These results agree with a previous comparative analytical study, which showed that endocannabinoids (AEA) increased in the retina from human ARMD eyes [27]. As a lipid soluble substance, AEA cannot be stored in vesicles; and therefore it is synthesized on demand and travels, in a retrograde direction, across the postsynaptic membrane to the presynaptic membrane, where it activates presynaptic CB1 and CB2 receptors [36]. After cellular uptake, AEA is degraded via the enzyme FAAH [14]. Variation of ECS also can be observed in other neurodegenerative disorders, such as Parkinson disease, Alzheimer disease, and multiple sclerosis [2,37,38]. The variation of ECS may be an endogenous response to maintain endocannabinoid homeostasis and regulate the pathologic function of neuron cells [39,40]. In line with the endocannabinoid homeostasis theory, there are now several examples of the successful use of ECS-directed drugs used to alleviate the clinical symptoms of neurodegenerative diseases in animal models [2,41,42]. These results suggest that cannabinoid receptors may be potential targets for therapeutic interventions for ARMD.

We also introduced CP55,940, which is a nonselective CB1/CB2 receptor agonist in oxidative stress-induced RPE cellular damage. We found that CP55,940 protected ARPE-19 cells from oxidative stress-induced cell damage and intracellular ROS generation in a dose-dependent way with excellent efficacy. We further explored whether CP55,940 induced cytoprotective signaling pathways while rescuing RPE cells from oxidative damage. The PI3K/Akt- and ERK1/2-mediated survival signal pathways had been suggested to protect RPE cells from oxidative stress [43,44]. We therefore addressed whether CP55,940 could modulate PI3K/Akt and ERK1/2 pathways, and found that CP55,940 could enhance H_2_O_2_-induced activation of PI3K/Akt and reduce the activation of the ERK1/2 pathway. We further investigated whether the selective activation of CB1 or CB2 receptor could protect RPE cells from oxidative stress induced damage. We used a cell viability assay to determine viability in cells treated with H_2_O_2_. We demonstrated that CB2 receptor agonist, but not CB1 receptor agonist, significantly protected human RPE cells from oxidative stress. Several studies have shown CB1 receptor activation to mediate neuroprotection, but this is not a universal finding [45,46]. This neuroprotective effect could be related to different cell types and pathological conditions.

In conclusion, we demonstrated the expression of CB1 and CB2 receptors and FAAH in human RPE cells, and their changes in oxidative stress conditions. The RPE cells perform vital functions for safeguarding photoreceptor cells against oxidative stress and are involved in the pathogenesis of ARMD [47]. These findings open up the attractive possibility that a correlation exists between endocannabinoid homeostasis and the onset of ARMD. However, as we observe inhibition, rather than the more usual stimulation, of ERK signaling, the involvement of receptors other than CB1 as well as CB2 in the effects of CP55,940 or JWH015 cannot be excluded. More studies using siRNA or specific CB1 and CB2 receptor antagonists should be performed to examine whether manipulating the levels of cannabinoid receptors could be a novel pharmacological approach to treat ARMD in the future.
